# Pulmonary hypertension in Behçet’s disease: echocardiographic screening and multidisciplinary approach

**DOI:** 10.55730/1300-0144.5617

**Published:** 2023-02-01

**Authors:** Berkan ARMAĞAN, Metin OKŞUL, Yusuf Ziya ŞENER, Alper SARI, Abdulsamet ERDEN, Gözde Kübra YARDIMCI, Kadir Mutlu HAYRAN, Levent KILIÇ, Ömer KARADAĞ, Ergün Barış KAYA, Sadberk Lale TOKGÖZOĞLU, Ali İhsan ERTENLİ, Ali AKDOĞAN

**Affiliations:** 1 Division of Rheumatology, Department of Internal Medicine, Faculty of Medicine, Hacettepe University, Ankara, Turkey; 2 Department of Cardiology, Faculty of Medicine, Hacettepe University, Ankara, Turkey; 3 Department of Preventive Oncology, Faculty of Medicine, Hacettepe University, Ankara, Turkey

**Keywords:** Behçet’s disease, pulmonary hypertension, pulmonary involvement, pulmonary arterial involvement, echocardiography

## Abstract

**Background/aim:**

Little is known about the prevalence and causes of pulmonary hypertension (PH) in Behçet’s disease (BD). This study was conducted to determine the prevalence and causes of PH in BD.

**Materials and methods:**

In this descriptive study, we screened 154 patients with BD for PH using transthoracic echocardiography between February 2017 and October 2017. An estimated systolic pulmonary arterial pressure (sPAP ≥ 40 mmHg) was used as the cut-off value to define PH. Patients with BD were categorized into 5 groups according to organ involvement including mucocutaneous/articular, ocular, vascular, gastrointestinal, and neurologic involvement. Additional laboratory and imaging results were obtained from hospital file records to determine the causes of PH.

**Results:**

PH was detected in 17 (11%) patients. Nine (52.9%) of these patients had group II PH (due to left heart disease), 4 (23.5%) had IV PH (due to pulmonary arterial involvement), and 1 had III PH (due to chronic obstructive lung disease). The frequency of PH was higher in BD patients with vascular involvement than those without (52.9% vs 28.5%; p = 0.04). Among 10 patients with pulmonary artery involvement (PAI) 4 (40%) had PH. Although the vascular BD group had the highest rate of PH, we observed no statistically significant difference in the frequency of PH between the predefined BD subgroups.

**Conclusions:**

PH is not rare in patients with BD. The majority of BD patients with PH are in group II or IV PH. Patients with vascular involvement carry a higher risk for the development of PH. Monitoring BD patients with PAI should be considered for the development of group IV PH.

## 1. Introduction

Behçet’s disease (BD) is a multisystem disorder classified as a variable vessel vasculitis, characterized by remitting and relapsing inflammatory attacks [1]. The most common manifestation are mucocutaneous lesions (up to 95%–100%) followed by ocular (50%), articular (50%), vascular (40%), gastrointestinal (up to 50% in Asian populations), and central nervous system (5%–10%) involvement [1–4].

Pulmonary hypertension (PH) is defined as a mean pulmonary artery pressure > 20 mmHg at right heart catheterization (RHC) performed at rest [5]. Regardless of its cause, PH is considered to be a poor prognostic factor. Transthoracic echocardiography (TTE) is the most frequently used tool for screening PH and a systolic pulmonary arterial pressure (sPAP) ≥ 40 mmHg measured by TTE is suggestive for the presence of PH [6]. Causes of PH are categorized into 5 groups; Group I: pulmonary arterial hypertension, Group II: PH due to left heart disease, Group III: PH due to lung disease and/or chronic hypoxia, Group IV: PH due to chronic thromboembolic pulmonary hypertension (CTEPH) or other pulmonary artery obstructions (i.e. arteritis), Group V: PH with unclear and/or multifactorial mechanisms [6].

Pulmonary arterial involvement (PAI), cardiac involvement and drugs (such as interferon-α) are the potential causes of PH in BD [2, 6, 7]. The frequency of PH in BD was reported between 9% and 65% using TTE in various studies. However, these studies were conducted with a small number of patients or in selected patient groups. Causes of PH were incompletely defined in some [2, 3, 8, 9]. The aim of this study is to determine the prevalence and causes of PH in a large series of BD.

## 2. Materials and methods

### 2.1. Patients

In this descriptive study, 154 adult patients with BD who presented to the Rheumatology Outpatient Clinic between February 2017 and October 2017 were consecutively included into the study. All patients fulfilled the International Study Group criteria for diagnosis of BD [10]. Age < 18 years and pregnancy were the exclusion criteria. Exclusion criteria did not include any drug use. The study protocol was approved by the local institutional ethics committee. All patients gave informed consent to participate in the study.

### 2.2. Study design

All subjects were evaluated by a detailed medical history and physical examination. All participants were thoroughly questioned about signs, symptoms and history of any vascular, cardiac or pulmonary involvement. Data regarding demographics, comorbidities, smoking status, disease duration, type of systemic involvement, previous and current medications and laboratory and imaging results were obtained from medical records and by face to face interview. Blood samples for haemoglobin and acute phase reactant levels were collected at the day of TTE evaluation. We classified BD patients into PH groups according to current guidelines by a multidisciplinary evaluation [6]. Patients with BD were categorized into 5 groups according to organ involvement: group 1: mucocutaneous and articular involvement, group 2: ocular involvement, group 3: vascular involvement, group 4: gastrointestinal involvement and group 5: neurologic involvement [11, 12].

### 2.3. Echocardiographic measurements

Echocardiographic measurements were performed by a cardiologist (MO) who was blinded to the clinical information. Complete 2-dimensional, M-Mode, and Doppler echocardiographic imaging were performed using a commercially available echocardiography device (Acuson Sequoia C256 and a 3.2 MHz transducer (Siemens Medical Solutions, Mountain View, CA, USA). Left ventricular (LV) end-systolic and end-diastolic diameters, septal and posterior wall end-diastolic thickness, right ventricular (RV) end-diastolic diameter were measured by 2D imaging and valvular functions were evaluated by colour Doppler. Left ventricular ejection fraction (LVEF) was calculated by the modified Simpson’s rule [13]. sPAP was calculated by the simplified Bernoulli equation from the tricuspid regurgitation jet velocity (V) by adding the estimated right atrial (RA) pressure (sPAP = 4(V)^2^ + RA pressure). RA pressure was determined by the inferior vena cava diameter in conjunction with respiratory changes. An estimated sPAP ≥ 40 mmHg was used as the cut-off value to define PH [6].

Left ventricular diastolic function was determined by using the recommendations of American Society of Echocardiography and European Association of Cardiovascular Imaging guidelines. Accordingly, LV diastolic dysfunction was determined based on early diastolic (mitral E peak) and late diastolic (mitral A peak) velocities of the mitral inflow, septal and lateral wall early diastolic velocities (e′), E/e′ ratio, tricuspid valve regurgitation velocity and left atrial volume index [14].

### 2.4. Statistical analysis

Statistical analysis was performed using SPSS version 23.0 (SPSS Inc., Chicago, USA). The variables were investigated using visual (histograms, probability plots) and analytical methods (Shapiro–Wilk’s test) to determine whether or not they are normally distributed. Continuous data were described as mean (standard deviation, SD) or median (minimum–maximum, min–max) and categorical variables as percentages. Chi-square or Fisher’s exact test was used to compare categorical variables. When comparing PH prevalence among groups, the statistical analyses were limited to patients with single organ involvement. Student’s *t*-test or Mann–Whitney U test was used to compare continuous variables. A p-value of <0.05 was considered significant.

## 3. Results

A total of 154 (40% female) patients with BD were enrolled into the study. The median (min–max) age and disease duration of the patients were 41 (18–73) years and 126 (6–540) months, respectively. Nine (5.8%) patients had diabetes mellitus (DM) and 26 (16.9%) patients had hypertension (HTN). Demographic and clinical characteristics of BD patients are summarized in Table 1. There were no patients with malignancy or breast feeding at the time of the evaluation.

Pulmonary hypertension was detected in 17 (11%) patients using TTE. There were no significant differences between BD patients with and without PH regarding age, sex, disease duration, presence of HTN and DM, serum haemoglobin, erythrocyte sedimentation rate and C-reactive protein levels as shown in Table 1. The frequency of PH was higher in BD patients with vascular involvement than those without (52.9% vs 28.5%; p = 0.04). Ten (6.5%) patients had PAI (1 with pulmonary artery aneurysm + pulmonary arterial thrombosis and 9 with pulmonary arterial thrombosis). Four (40%) of the patients with PAI had PH. PAI was more frequent in patients with PH than in those without (23.5% vs 4.4%; p = 0.003). There was no statistically significant difference between the predefined BD groups regarding the percentage of PH as shown in Table 2. There were no PH cases among patients with isolated gastrointestinal or neurologic involvement.

The causes of PH in BD patients were; Group II PH in 9 (52.9%), Group IV PH in 4 (23.5%), and Group III PH in 1(5.9 %). We could not define the exact cause of the PH in 3 patients. Only 9 (52.9%) of the patients with PH were symptomatic (New York Heart Association functional capacity (NYHA FC) >1). Clinical features of the patients with PH are presented in Table 3. Left ventricular diastolic dysfunction (in 7 patients) and valvular heart disease (in 4 patients) were the causes of group 2 PH. At the time of their evaluation, all the patients with group IV PH had pulmonary vascular occlusive lesions and were on immunosuppressive and/or interferon-α (IFN-α) treatment. Three out of 4 patients with group IV PH were symptomatic.

Left ventricular diastolic dysfunction was found in 32 (20.8%) patients in the whole BD group. Only 1 patient had isolated LV systolic dysfunction. The percentage of LV diastolic dysfunction was significantly higher in patients with PH than in those without (47.1% vs 17.6%; p = 0.005). Patients with LV diastolic dysfunction were older (49.5 ± 11.7 vs 39.8 ± 12.0; p < 0.0001) and had more frequent HTN [10 (31.3%) vs 16 (13.2%); p = 0.016] than those without diastolic dysfunction.

Sixty-seven (43.5%) patients were either currently under IFN-α therapy or received IFN-α in the past. There was no significant difference between the patients with or without IFN-α therapy regarding the mean sPAP (30.3 ± 6.4 vs. 29.6 ± 6.5 mm Hg; p = 0.448). The rate of PH was similar between these groups [10.4% (n = 7) vs 11.5% (n = 10); p = 0.837].

## 4. Discussion

In this study, PH frequency was 11% in BD patients. Group II (52.9%) and Group IV (23.5%) PHs were the major causes of PH. In the whole group with PH, 52.9% of patients were symptomatic, whereas in patients with group IV PH, 3 out of 4 (75%) were symptomatic.

There is only one study in the literature which enrolled 77 BD patients where PH was systematically investigated [3]. In that study, the frequency of PH in BD was reported as 9% with echocardiographic evaluation. The authors underlined that elevated sPAP was present only in patients with vascular involvement; mainly in patients with PAI. They stated that most of the patients with PH had mild sPAP elevations. On the other hand, they did not define the causes of PH in their patient group individually [3]. In that study, they used a group of patients with systemic sclerosis as a control group in whom PH is frequent. Interestingly, the frequency of elevated sPAP was found to be similar between BD patients with PAI and systemic sclerosis. The frequency of PH was also reported to be higher in BD as compared to controls in the small study by Heperet al. [15]. In BD patients with PAI, the frequency of PH was reported to be 65% at initial evaluation in one study and 11% in another. Moreover, the presence of PH was found as a poor prognostic factor in patients with PAI [2, 8]. In our study, overall PH frequency was 11%, whereas PH frequency was 40% in patients with PAI. Only half of our patients were symptomatic. The frequency of PH was significantly higher in patients with vascular involvement as compared to those without vascular involvement. These results are consistent with the literature. However, although the vascular BD group had the highest rate of PH, we observed no statistically significant difference in the frequency of PH between the predefined 5 disease groups. PH was not observed in patients with single gastrointestinal or neurological involvement.

Left heart diseases associated with PH (group II PH) is considered to be the most common cause of PH in general [6]. In the present study, group II PH was also the leading cause of PH in BD patients. Most of the patients with group II PH had LV diastolic dysfunction or/and valvular heart disease. Previously, many different cardiac abnormalities have been reported to be associated with BD including LV diastolic dysfunction and valvular heart diseases [16, 17]. LV diastolic dysfunction was present in 20.8% of our patients and the rate of LV diastolic dysfunction was higher in patients with PH as compared to patients without PH. LV diastolic dysfunction is a frequent cause of PH and the prevalence of LV diastolic dysfunction increases by age and with the presence of HTN [18]. As expected, in our study, patients with LV diastolic dysfunction were older and had HTN more frequently, as compared to patients without LV diastolic dysfunction. Therefore, it is hard to conclude that PH due to left heart disease is secondary to BD in all patients, based on our findings. Similarly, it can be very challenging to determine the definite cause of a valvular heart disease in the presence of BD. Regardless of their cause, all disorders associated with the left heart including LV diastolic dysfunction and heart valve diseases can contribute to the development of PH in BD as in the general population.

**Figure 1:**
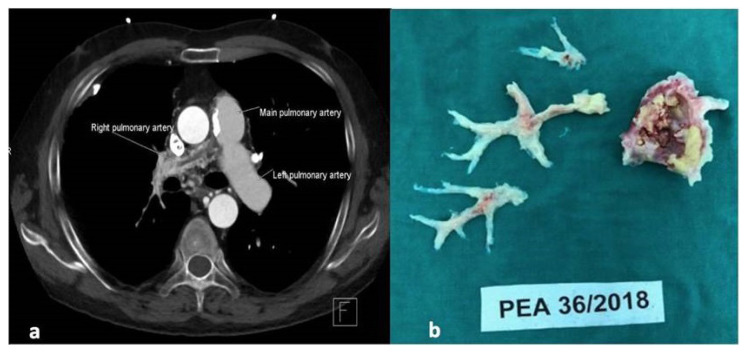
Image of patient no: 3 with group IV pulmonary hypertension (**a**) complete occlusion of the right pulmonary artery and (**b**) specimen removed from lung during pulmonary endarterectomy.

Pulmonary arterial involvement is one of the major causes of mortality in BD [1]. Pulmonary arterial aneurysms and pulmonary arterial thromboses are the most common lesions and they tend to be multiple [3]. Although deep venous thrombosis is a common manifestation of BD, pulmonary thromboembolism is rare [2, 19]. In this study, patients with PAI had PH more frequently as compared to patients without PAI. There were 4 patients with group IV PH due to PAI. Three of the four patients with group IV PH were symptomatic. Theoretically, these 3 patients were candidates for endarterectomy operation. Steroids and immunosuppressive agents are highly effective for controlling disease activity in BD [20]. Disappearance or regression of pulmonary arterial aneurysms and thrombotic lesions has been demonstrated in many patients with only immunosuppressive treatment. On the other hand, despite adequately controlled disease activity, Behçet’s lesions resolve slowly and the presence of residual sequelae lesions can be documented with different imaging methods [2]. Unresolved or organized thromboses due to defective fibrinolysis are considered to be one of the main mechanisms in the development of CTEPH [21, 22]. Long standing sequelae pulmonary vascular lesions may trigger a similar process in BD and can lead to PH. Endarterectomy operations have been suggested to be a successful treatment option in BD patients with PH. Authors reported that only one out of nine patients with PH died after endarterectomy operation [23]. On the other hand, complication rate of surgical procedures is higher in patients with vasculitic syndromes, especially during the active phase of the disease. Vascular surgery can cause a flare in BD by triggering a vasculitic process, similar to pathergy reaction [24, 25]. Moreover, involvement of all the arterial layers is typical in BD which is different from CTEPH that mainly affects the intima [21, 22, 26]. Data about the clinical course of the BD patients with group IV PH is limited. Therefore, defining patients who are suitable for endarterectomy operation is challenging. Endarterectomy may be an effective treatment option in patients with group IV PH due to sequelae pulmonary vascular lesions despite well controlled disease activity. Disease activities of all the 4 patients with group IV PH in our study were under control. We decided to follow up 2 mildly symptomatic and 1 asymptomatic patients by echocardiography after discussing their clinical conditions with cardiologists, cardiovascular surgeons, and the patients. We performed right heart catheterization only for the patient in whom we performed surgical endarterectomy. This symptomatic patient had a long disease duration (>3 years) and a severe chronic proximal right pulmonary artery occlusive lesion unlikely to respond to immunosuppressive treatment. The radiological image of this patient is shown in Figure 1a, and the surgically removed pulmonary endarterectomy specimens are shown in Figure 1b.

Interferon α is an effective treatment agent in BD [20]. Both interferon α and β are in the list of possible drugs that can cause PH [5]. Elevated endothelin levels were found in patients with PH receiving IFN-α therapy for chronic hepatitis C and systemic sclerosis [27]. Most of the patients in the literature who are on IFN-α treatment and developed PH also had other risk factors for the development of PH such as portal hypertension or human immunodeficiency virus infection. The authors suggested that IFN-α treatment can be a trigger in patients who are prone to develop PH [28]. In this study, we did not document any association between IFN-α treatment and PH. Our patients with PH who were under IFN-α treatment had all well-defined clinical conditions to explain the presence of PH other than IFN-α effect. However, as there is no satisfactory data about the effect of IFN-α treatment on PH development in BD, patients who are on this treatment should be followed up carefully.

There were some limitations to consider in our study. We used only sPAP of the patients to define PH and did not regularly perform RHC to confirm the diagnosis. Although most of our patients had clinical conditions compatible with the presence of PH, this might have increased the prevalence of PH in our study. We could not compare the characteristics of BD patients among different PH groups in detail due to the limited number of subjects. Previous studies suggested that PH could be associated with disease activity in systemic lupus erythematosus and Takayasu arteritis [29, 30]. We did not investigate the association between disease activity and the presence of PH. Lastly, the absence of a control group in our study can be considered another limitation.

We documented that PH is not rare in BD patients. The majority of BD patients with PH are in group II or IV PH. All BD patients suspected of having PH should be carefully investigated for the presence of left heart disease. Monitoring BD patients with PAI should be considered for the development of group IV PH.

## Figures and Tables

**Table 1:** Demographic and clinical features of Behçet’s disease patients.

	All patients (n = 154)	sPAP ≥ 40 mm Hg (n = 17)	sPAP < 40 mm Hg (n = 137)
**Female, n (%)**	62 (40.3)	6 (35.3)	56 (40.9)

**Age, median (min–max)**	41 (18–73)	46 (24–72)	40 (18–73)

**Disease duration (months), median (min–max)**	126 (6–540)	168 (12–540)	120 (6–480)

**Diabetes mellitus, n (%)**	9 (5.8)	1 (5.9)	8 (5.8)

**Hypertension, n (%)**	26 (16.9)	5 (29.4)	21 (15.3)

**Smoking, n (%)**			
**Never smoker**	76 (49.4)	7 (41.2)	69 (50.4)
**Current smoker**	47 (30.5)	5 (29.4)	42 (30.7)
**Ex-smoker**	31 (20.1)	5 (29.4)	26 (19.0)

**Oral ulcer, n (%)**	154 (100)	17 (100)	137 (100)

**Genital ulcer, n (%)**	104 (67.5)	11 (64.7)	93 (67.9)

**Erythema nodosum, n (%)**	64 (41.6)	7 (41.2)	57 (41.6)

**Papulo-pustular lesion, n (%)**	35 (22.7)	3 (17.6)	32 (23.4)

**Acneiform lesions, n (%)**	105 (68.2)	8 (47.1)*	97 (70.8)*

**Articular involvement, n (%)**	35 (22.7)	4 (23.5)	31 (22.6)

**Uveitis, n (%)**	75 (48.7)	9 (52.9)	66 (48.2)

**Pathergy, n (%)**	40 (26)	6 (35.3)	34 (24.8)

**Vascular involvement, n (%)**	48 (31.2)	9 (52.9)**	39 (28.5)**

**Pulmonary arterial involvement, n (%)**	10 (6.5)	4 (23.5)***	6 (4.4)***

**Neurologic involvement, n (%)**	18 (11.7)	2 (11.8)	16 (11.7)

**Gastrointestinal involvement, n (%)** +	12 (7.8)	1 (5.9)	11 (8.0)

**Haemoglobin (g/dL), mean** ± **SD**	13.8 ±1.4	13.8±1.3	13.8±1.4


**Erythrocyte sedimentation rate (mm/h), median (min–max)**	10 (2–65)	8 (2–53)	10 (2–65)

**C-reactive protein (mg/dL), median (min–max)**	0.47 (0.1–15.8)	0.33 (0.19–7.9)	0.48 (0.1–15.8)

sPAP: Systolic pulmonary artery pressure, min: minimum, max: maximum.

+ Including patients with Budd Chiari syndrome.

* p = 0.04,

** p = 0.04,

*** p = 0.003.

**Table 2:** Distribution of pulmonary hypertension according to Behçet’s disease groups.

A. BD groups+	Mucocutaneous and articular+ (n = 39)	Ocular (n = 75)	Vascular (n = 48)	Gastrointestinal++ (n = 12)	Neurologic (n = 18)	
**sPAP ≥ 40, n (%)**	2 (5.1)	9 (12)	9 (18.8)	1 (8.3)	2 (11.1)	
**B. BD Groups with single organ involvement**	**Mucocutaneous and articular (n = 39)**	**Ocular (n = 47)**	**Vascular (n = 23)**	**Gastrointestinal (n = 1)**	**Neurologic (n = 9)**	**p** *
* **sPAP ≥ 40, n (%)**	2 (5.1)	4 (8.5)	5 (21.7)	0	0	0.246

BD: Behçet’s disease, sPAP: Systolic pulmonary artery pressure.

+ All the groups may have other organ/system involvements except mucocutaneous and articular group,

++ including patients with Budd Chiari syndrome.

* Fisher’s exact test for correction.

**Table 3:** Clinical characteristics of Behçet’s disease patients with pulmonary hypertension*.

Patient no.	Age, sex	Disease duration (months)	BD clusters	Risk factor for PH prior to TTE	NYHA FC	Echocardiographic findings	Lung imaging	Classification of PH
**1**	67, female	480	Vascular Neurologic	DM, HT, DVT	II	sPAP 50 mmHg, E<A, LVDD grade I Grade 2 MR	No pulmonary arterial lesions	II
**2**	54, female	312	Vascular Ocular	PAI, IFN	I – II	sPAP 50 mmHg E<A, LVDD grade I	Pulmonary arterial lesions	IV
**3**	49, male	192	Vascular	PAI Cardiac thrombosis IFN	III	sPAP 50 mmHg, RHC: PAP 50/32/20 mmHg	Pulmonary arterial lesions	IV
**4**	57, male	324	Ocular Neurologic	IFN	I – II	sPAP 45 mmHg, rade 2 AR and MR	-	II
**5**	41, male	114	Ocular	None	I	sPAP 45 mmHg	No pulmonary arterial lesions	?
**6**	65, male	444	Vascular Ocular	COPD (heavy smoker), HT, DVT	II	sPAP:45 mmHg Grade 2 MR	Severe emphysema No pulmonary arterial lesions	III
**7**	70, female	540	Ocular	HT, CKD Cardiac valvular disease	II	sPAP 45 mmHg AVR, grade 2 AR E<A, LVDD grade III	-	II
**8**	33, male	48	Vascular	PAI Cardiac thrombosis IFN	II	sPAP 45 mmHg, RV apex, RV thrombus 16×18 mm	Pulmonary arterial lesions	IV
**9**	54, female	84	Ocular	CAD, IFN	I	sPAP 40 mmHg E<A, LVDD grade I	-	II
**10**	40, male	168	Vascular Ocular	PAI DVT, IFN	I	sPAP 40 mmHg	Pulmonary arterial lesions	IV
**11**	46, male	192	Ocular Gastrointestinal	None	I	sPAP 40 mmHg, E<A, LVDD grade I	No pulmonary arterial lesions	II
**12**	37, male	24	Vascular	DVT	I	sPAP 40 mmHg, E<A, LVDD III	No pulmonary arterial lesions	II
**13**	24, male	72	Ocular	None	I	sPAP 40 mmHg, E<A, LVDD III	-	II
**14**	31, female	96	Mucocutaneous and articular	Anaemia	I	sPAP 40 mmHg E<A, LVDD grade III	-	II–V
**15**	72, female	348	Mucocutaneous and articular	HT	II	sPAP 40 mmHg	-	?
**16**	33, male	12	Vascular	SVT, (EPS-RFA) IFN	II	sPAP 40 mmHg Grade 2 AR	No pulmonary arterial lesions	II
**17**	46, male	120	Vascular	None	I	sPAP 40 mmHg minimal pericardial effusion	No pulmonary arterial lesions	?

* Patients were listed according to their sPAP values from highest to lowest.

BD: Behçet’s disease, PH: Pulmonary hypertension, TTE: Transthoracic echocardiography, NYHA FC: New York Heart Association functional class, LVDD: Left ventricular diastolic dysfunction, DM: Diabetes mellitus, HT: Hypertension, DVT: Deep venous thrombosis, sPAP: Systolic pulmonary arterial pressure, MR: Mitral regurgitation, PAI: Pulmonary arterial involvement, IFN: Interferon α, RHC: right heart catheterization, PAP: Pulmonary arterial pressure, AR: Aortic regurgitation, COPD: Chronic obstructive pulmonary disease, CKD: Chronic kidney disease, AVR: Aortic valve replacement, RV: Right ventricle, CAD: Coronary artery disease, SVT: Supraventricular tachycardia, EPS: Electrophysiology study, RFA: Radiofrequency ablation.
